# Disparities in long-lasting insecticidal bed net usage and malaria burden 2 years after a mass distribution campaign in central Côte d’Ivoire: A cross-sectional survey prior to a cluster randomised trial

**DOI:** 10.1371/journal.pgph.0005642

**Published:** 2026-01-22

**Authors:** Colette Sih, Serge B. Assi, Edouard Dangbenon, Benoit Talbot, Joseph Biggs, Alphonsine A. Koffi, Ludovic P. Ahoua Alou, Louisa A. Messenger, Marius Gonse Zoh, Soromane Camara, Manisha A. Kulkarni, Natacha Protopopoff, Raphael N’Guessan, Jackie Cook

**Affiliations:** 1 Faculty of Epidemiology and Population Health, Department of Infectious Disease Epidemiology, London School of Hygiene and Tropical Medicine, London, United Kingdom; 2 Institut Pierre Richet (IPR)/Institut National de Santé Publique (INSP), Bouaké, Côte d’Ivoire; 3 School of Epidemiology & Public Health, Faculty of Medicine, University of Ottawa, Ottawa, Ontario, Canada; 4 Faculty of Infectious and Tropical Diseases, Disease Control Department, London School of Hygiene and Tropical Medicine, London, United Kingdom; 5 Department of Environmental and Occupational Health, School of Public Health, University of Nevada, Las Vegas, Nevada, United States of America; 6 Health Interventions Unit, Department of Epidemiology and Public Health, Swiss Tropical & Public Health Institute, Allschwill, Switzerland; 7 University of Basel, Basel, Switzerland; 8 Medical Research Council (MRC) International Statistics and Epidemiology Group, London School of Hygiene and Tropical Medicine, London, United Kingdom; Tulane University School of Public Health and Tropical Medicine, UNITED STATES OF AMERICA

## Abstract

Identifying and tackling inequities in long-lasting insecticidal bed net (LLIN) coverage and usage is key in reducing malaria burden. This baseline study, prior to an LLIN trial, describes factors associated with LLIN usage and malaria infection prevalence, two years after a mass distribution in Côte d’Ivoire. In July 2023, cross-sectional data were obtained from randomly selected individuals of all ages in each of 33 study clusters, capturing information on socio-economic status, LLIN ownership, usage and results of malaria rapid diagnostic tests. Random-effects multivariable logistic regression analyses were used to assess factors associated with LLIN usage and malaria infection. A total of 1,672 participants were recruited. LLIN ownership and access were 66.3% (95% CI: 59.4-73.2) and 27.8% (95%CI: 22.6-33.1), respectively. LLIN usage was 50.0% (adjusted odds ratio [aOR]: 0.50; 95%CI: 0.34-0.75), 44.3% (aOR: 0.41; 95%CI: 0.26-0.62) and 56.9% (aOR: 0.72; 95%CI: 0.53-0.98) in participants aged, 5–9, 10–14 and ≥15years, respectively, compared to 63.2% in under-fives. LLIN usage was lowest in females aged 10–14 years (41.0%) and highest in under-five males (68.9%). The odds of LLIN usage were lowest in the second (aOR: 0.63; 95%CI: 0.44-0.90) and middle wealth quintile (aOR: 0.69; 95%CI: 0.47-1.00) compared to the poorest quintile (58.2%). Malaria infection prevalence was 41.1% (95%CI: 37.2-45.0). When compared to under-fives with a malaria prevalence of 61.3%, 5–9, 10–14 and ≥15years had 75.0% (aOR: 1.86; 95%CI: 1.24-2.78), 67.0% (aOR:1.18; 95%CI: 0.78-1.80) and 23.0% (aOR: 0.17; 95%CI: 0.12-0.23) malaria prevalence, respectively. Males (75.8%) and females (74.1%) aged 5–9 years had the highest malaria risk. LLIN users had an infection prevalence of 38.5% (aOR: 0.74; 95%CI: 0.58-0.94), compared to 44.3% in non-users. School-based net distributions, malaria education, routine screening and treatment and chemoprophylaxis in schools alongside community sensitization campaigns are recommended to improve protection and reduce infection risk among school-aged children.

## Introduction

Malaria remains a major global public health problem, causing an estimated 249 million cases and 608,000 deaths in 2022, of which 94% occurred in the WHO African Region [1]. Within Africa, West Africa carries a disproportionately high burden, driven by high transmission intensity and widespread insecticide resistance in local vectors. Côte d’Ivoire ranks among the fifteen high-burden, high-impact countries that together account for approximately 70% of global malaria cases and deaths. In 2023, the country contributed about 2.8% of global malaria cases and deaths [1]. Côte d’Ivoire is a malaria-endemic country, with perennial transmission, peaking during the rainy season, and malaria remains the leading cause of morbidity and mortality in children under the age of five years [2–4]. The national malaria control strategy emphasizes universal coverage of vector control interventions—particularly long-lasting insecticidal nets (LLINs)—alongside improved case management and preventive treatment in pregnancy [2–4].

Despite large-scale LLIN distributions, malaria burden remains high, reflecting both biological and social determinants of inequity [5,6]. Pyrethroid resistance has greatly threatened progresses made in malaria control. In response, next-generation LLINs combining pyrethroids with other active ingredients such as piperonyl butoxide (a synergist) or chlorfenapyr (an adulticide) have been developed and are used routinely in several countries, including Cote d’Ivoire [7–9]. Equity—defined as fair access to malaria prevention and treatment regardless of socio-economic status, sex, or age—is central to achieving universal health coverage [5]. Yet, differences in LLIN use persist. Studies have shown that LLIN usage tends to be lower among school-aged children and in households with middle or higher income levels [10–12]. Gender differences are also observed: men are often at higher occupational risk due to evening outdoor activities [13–15], while women may face barriers to care-seeking and exposure during household chores at peak mosquito-biting hours [16,17]. These inequities contribute to sustained malaria transmission and limit the impact of control interventions [6].

To date, few studies in Côte d’Ivoire have systematically examined disparities in LLIN usage and their association with malaria infection. Understanding these baseline patterns is critical to identify population groups that remain underserved and to inform more equitable net distribution strategies. Hence, this study aimed to describe disparities in LLIN usage, and factors associated with net usage and malaria infection prevalence, using baseline data collected prior to a cluster-randomised trial evaluating chlorfenapyr-pyrethroid and piperonyl butoxide-pyrethroid LLINs in central Côte d’Ivoire.

## Materials and methods

### Study design and setting

**Fig 1:**
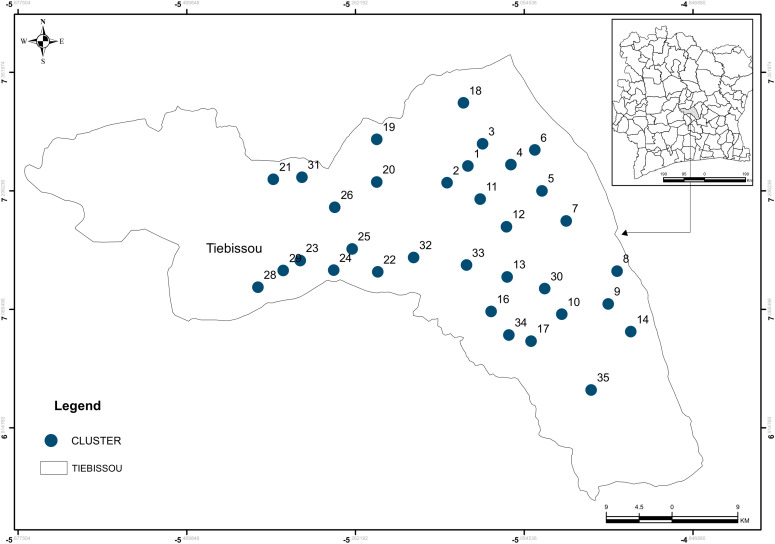
Map of the study area and clusters.

This community-based cross-sectional study was conducted between the 18 and 28 July 2023 in 41 villages grouped into 33 study clusters (each cluster consisting of 1–3 villages) in Tiebissou department, Gbêkê region, Lacs district, central Côte d’Ivoire (Fig 1). The survey served as the baseline for a three-arm cluster-randomised controlled trial evaluating the efficacy of next-generation LLINs [18].

The study area has an estimated population of 116,321 spread over 2,410 km^2^ [19]. It’s tropical climate is characterised by two rainy (May–July and October–November) and two dry (December–April and August–September) seasons, with malaria transmission peaking in May to November. The primary malaria vectors, *Anopheles gambiae s.l.*, *An. coluzzii*, and *An. funestus s.l*., show intense resistance to pyrethroids [3]. According to the 2021 Demographic and Health Survey (DHS), malaria prevalence in children aged 6–59 months in Côte d’Ivoire was 51.3% [2].

Vector control activities are primarily based on mass LLIN distribution campaigns every three years, complemented by continuous distribution through antenatal and paediatric consultations. The most recent mass campaign performed by the National Malaria Control Programme (NMCP), held 11 March to 5 May 2021, distributed PermaNet®2.0 (blue polyester net coated with deltamethrin) at a ratio of one LLIN for every two persons (based on household size), with no maximum cap per household. In 2021, household LLIN ownership, access, and usage were estimated at 76.7%, 52.5%, and 55.3%, respectively, in the Lacs district [2].

### Study population and sampling

A census performed from June 8–27, 2023 identified all households and residents within the study area [18,20]. Based on sample size calculations for the main trial, a minimum of 50 participants per cluster were required to achieve the desired power for detecting differences in malaria infection prevalence between each intervention arm and the control arm, while accounting for cluster sampling.

Participants were randomly selected from census lists. By chance, more than one individual could be selected from the same household; when this occurred, a maximum of two participants per household was permitted to avoid excessive clustering. Eligible participants were residents of the study area for at least three months who provided written informed consent (or parental consent/child assent as appropriate). Severely ill individuals were not enrolled but referred to the nearest health facility. Vacant or unfound dwellings were recorded and replacements were drawn to maintain target sample size.

### Data collection and analysis

Two structured, interviewer-administered questionnaires were used: 1/household questionnaire which captured socio-demographic data on all household members, household possessions, dwelling characteristics, and the number, type, and origin of bed nets; and 2/the individual questionnaire administered to each randomly selected participant to collect data on recent symptoms of malaria, infrared frontal temperature, and malaria test results. Temperature was used to identify fever (temperature ≥37.5°C) to provide additional insight into the diagnostic value of fever as a proxy indicator of malaria in the study population. Interviews were conducted primarily with the head of household or a designated representative in their absence. Malaria rapid diagnostic test (RDT) (STANDARD Q Malaria P.f/Pan Ag Test, SD Biosensor Inc., Republic of Korea; Catalogue number: 09MAL30D) was performed for all selected participants, irrespective of symptoms. All data were collected using Open Data Kit installed on tablets. All consents, interviews, temperature measurements and RDTs were performed by trained study nurses.

Data analyses were done with STATA/SE version 18.0 (Stata Corp LP, College Station, TX). A description of the exposure and outcome variables is shown in Table 1.

**Table 1:** Description of study variables.

Exposure variables	Variable description
Age group	Grouped into 4 categories: < 5, 5–9, 10–14 and ≥15 years
Sex	binary variable (male/female)
Socio-economic status	a household-level wealth index was calculated using principal component analysis, and then divided into quintiles (with quintile 1 being the poorest and quintile 5 the richest) and assigned to each study participant. The following variables were re-categorized into binary variables and included in the principal component analysis: principal income-generating activity and level of education of the head of the household, access to electricity and portable water, type of toilet, type of building materials (floor, roof and wall), asset ownership (bicycle, motorcycle, fan, television, radio, fridge, telephone, solar panel, solar-powered lamp, bed, mattress, sheep, and other livestock), and household overcrowding (based on the proportion of household size to the number of sleeping units).
Religious affiliation	Collected as a categorical variable and recoded into 4 groups: Christian, traditional beliefs, none and others.
Altitude	2 categories around the median: 153–214 metres (very low altitude) and 215–258 metres (low altitude)
Population density	Divided into 2 groups based on the median: 40–132 persons per square kilometre (very low) and 133–505 persons per square kilometre (low)
Normalized Difference Vegetation Index	Categorized into 3 groups with the following cut-offs: low (71–6464), moderate (6465–7765), and high (7766–9000) vegetation cover
Closest distance between household and health facility	Classified into 3 groups with the following cut-offs: 0.01-0.6km, 0.7-5.1km and 5.2-13.4km.
Nearest distance between household and lake	Classified into 3 groups with the following cut-offs: 0.4-5.0km, 5.1-8.6km and 8.7-36.8km.
**Outcome variables**	
Household LLIN ownership	Proportion of households with at least one LLIN.
Household LLIN access (based on 1 LLIN per 2 persons)	Proportion of households with at least one LLIN for every 2 people.
Population LLIN usage	Proportion of study participants who reported sleeping under an LLIN the previous night.
Malaria infection prevalence	Proportion of malaria positive infections on RDT out of the total number of RDTs performed, irrespective of the presence or absence of clinical signs and symptoms.

LLIN: long-lasting insecticidal net; RDT: rapid diagnostic test.

Continuous variables were summarized using means (and standard deviations [SD]) if normally distributed or medians (and interquartile ranges [IQR]) if skewed. Proportions were used to summarise binary and categorical variables. Missing data was minimal and no multiple imputation was performed.

To explore factors associated with LLIN usage and malaria infection prevalence, we conducted a risk factor analysis. Because the data were obtained from individuals nested within clusters, observations within the same cluster were likely to be correlated. To account for this non-independence and to obtain valid standard errors, logistic regression models with random intercepts for clusters were used. Univariate random-effects logistic regression models were first fitted to quantify the crude association between each socio-demographic variable and the outcome. Variables with a *p*-value <0.2 in likelihood ratio tests were considered for inclusion in the multivariable models. Known determinants of malaria risk and LLIN use—age, sex, and SES (derived through principal component analysis as described in Table 1)—were included a priori as confounders in the final models regardless of statistical significance. A backward stepwise selection approach was used, retaining a priori confounders and variables with a p-value <0.05 (based on likelihood ratio tests) in the final multivariable models. Interaction terms between sex and SES, and between age and sex, were tested based on prior evidence suggesting that the relationship between malaria risk and gender may vary by socio-economic position, with poorer women being disproportionately affected [17].

Geographic coordinates (datum: WSG84) of health facilities and selected households were uploaded into QGIS v3.30.3. The Earth Explorer database from United States Geological Survey was accessed to obtain altitude data (Global Multi-resolution Terrain Elevation Data 2010 [21]) and normalized vegetation cover data (EROS Visible Infrared Imaging Radiometer Suite [22]). The WorldPop database was used to obtain population density data [23]. The ESRI database, initially created by Messager *et al.* [24], was accessed to obtain the map outlines of all lakes. All coordinates were projected to UTM30N. Euclidean distances between each household and its nearest health facility and nearest lake in meters were calculated using the ‘Distance to nearest hub’ tool. Altitude, normalized vegetation cover and population density of each selected household was estimated using the ‘Sample raster values’ tool.

### Ethical statement

The trial received ethics approval from the Ministry of Health of Ivory Coast’s institutional review board (Ref: 002–23/MSHPCMU/CNESVS-km) and the institutional review board of London School of Hygiene and Tropical Medicine (Ref: 28390). Written informed consent was obtained from all hamlet leaders, household heads or their adult representatives and study participants. For minor participants (under the age of 18), written informed consent was obtained from their parents or legal guardians, as well as written assent obtained from minors aged over 10 years. Additional information regarding the ethical, cultural, and scientific considerations specific to inclusivity in global research is included in the Inclusivity in global research questionnaire.

## Results

### Description of the study population

A total of 1,672 participants from 1,653 households (with a total of 7,630 inhabitants) were recruited for this study, with a response rate of 99.3% (12 refusals). The median household size was 4 people (IQR:3–6). The median distance from household to the closest health facility and the nearest lake were 3.7km (IQR: 0.4- 6.4) and 7.7km (IQR: 4.1-10.6) respectively. The median altitude from sea level was 214m (IQR: 192–235). The median population density was 132 people/km^2^ (IQR: 96–245) and vegetation cover was moderate at 7,454 (IQR: 5,701–8,004).

Characteristics of the study population are shown in Table 2. Slightly over half of study participants were females (51.5%), with under-fives, 5–9, 10–14 and ≥15 year olds representing 15.6%, 13.9%, 11.1% and 59.4% of the population, respectively. About two-thirds of study participants were Christians. Almost all study participants (97.0%) reported going indoors before 2200h, 80.3% reported going to bed before 2200h the previous night and 46.6% of doors were closed after 2200h. About one-fifth (21.5%) of study participants reported using other insecticidal products against mosquitoes, such as insecticide sprays and coils. Thirty-one (1.9%) households were completely screened. Potential mosquito breeding sites were observed around 86.6% of households. Slightly over half of the household heads thought that malaria posed a serious problem in their community (n = 959; 58.0%), and about two-thirds thought that there were a lot of mosquitoes in their houses (n = 1,002; 60.6%).

**Table 2:** Characteristics of households and study participants enrolled in this study.

General characteristics		n (%)
Number of households		1,653 (100)
Ethnicity	Baoulé	1,624 (98.2)
	Other	29 (1.8)
Religion	Christianity	991 (60.0)
	Traditional religion	273 (16.5)
	None	311 (18.8)
	Other	78 (4.7)
**Head of household**		
Marital status	Single	384 (23.2)
	Married	1,126 (68.1)
	Separated/divorced	19 (1.2)
	Widow/widower	124 (7.5)
Education*	None	1,008 (61.0)
	Primary education	373 (22.6)
	Secondary and higher education	270 (16.4)
Occupation	Other	271 (16.4)
	Farming	1,382 (83.6)
**Household characteristics**		
Household size	1-3	607 (36.7)
	4-6	763 (46.2)
	7-30	283 (17.1)
Number of sleeping units	1-2	1,085 (65.6)
	3-4	456 (27.6)
	5-15	112 (6.8)
Number of LLINs per household	None	557 (33.7)
	1-2	820 (49.6)
	3-4	213 (12.9)
	>4	63 (3.8)
Window screening	No	1,614 (97.6)
	Yes	31 (1.9)
	Partially	8 (0.5)
Presence of potential mosquito breeding sites around dwelling	Yes	1,431 (86.6)
Other insecticidal products used against mosquitoes	Yes	356 (21.5)
**Household members**		
Number of household members		7,637 (100)
Sex	Male	3,726 (48.8)
	Female	3,911 (51.2)
Age group (in years)	<5	1,054 (13.8)
	5-15	2,257 (29.6)
	>15	4,326 (56.6)
Pregnancy status (amongst women >15 years)	Pregnant	94 (4.1)
**Study participants**		
Number of persons recruited		1,672 (100)
Sex	Male	811 (48.5)
	Female	861 (51.5)
Age group (in years)	<5	261 (15.6)
	5-9	232 (13.9)
	10-14	185 (11.1)
	≥15	994 (59.4)
Level of education	Not of school age	233 (13.3)
	None	757 (45.3)
	Primary education	408 (24.4)
	Secondary and higher education	284 (17.0)
Time participants went inside the house the previous night	Before 2200h	1,622 (97.0)
	After 2200h	41 (2.5)
	Don’t know	9 (0.5)
Time participant went to bed the previous night	Before 2200h	1,342 (80.3)
	After 2200h	303 (18.1)
	Don’t know	27 (1.6)
Time doors were closed the previous night	Before 2200h	670 (40.1)
	After 2200h	779 (46.6)
	Don’t know	223 (13.3)

*2 missing values.

### Household LLIN ownership and access

A total of 2,222 LLINs were counted, with 92.6% directly observed, 88.6% in current use and 88.3% hung-up. There was a median of 1 (IQR: 0–2) LLIN per household. A pyrethroid-only (deltamethrin) LLIN, PermaNet®2.0, was the most common LLIN (65.7%), with 75.8% of LLINs obtained during the mass distribution campaign of 2021 (Table 3).

**Table 3:** Characteristics of all long-lasting insecticidal nets present within households.

Characteristics		N (%)
**All long-lasting insecticidal nets present within households (N = 2,222)**		
Net type	PermaNet®2.0	1,461 (65.7)
	Interceptor®G2	337 (15.2)
	DuraNet®	126 (5.7)
	MAGNet®	95 (4.3)
	PermaNet®3.0 (PBO)	43 (1.9)
	Olyset Net®	18 (0.8)
	Yorkool®	17 (0.8)
	Dawa Plus®2.0	7 (0.3)
	Others*	33 (1.5)
	Don’t know	85 (3.8)
Duration of ownership	<1 year	63 (2.8)
	1-3 years	1,775 (79.9)
	3-4 years	266 (12.0)
	>4 years	118 (5.3)
Origin of nets	Last mass bed net campaign	1,685 (75.8)
	Antenatal care visit	216 (9.7)
	Routine immunization	177 (8.0)
	Non-profit organization	28 (1.3)
	Pharmacy	2 (0.1)
	Research institution	1 (0.1)
	Don’t know	86 (3.8)
	Other**	27 (1.2)
LLIN in current use	Yes	1,969 (88.6)
Reason why nets are not used (N = 243)	Reserved for future use	113 (44.7)
	Bed net not usable (too old or torn)	64 (25.3)
	Not enough sleeping spaces	16 (6.3)
	Enough bed nets for the household	10 (3.9)
	User definitely absent	8 (3.2)
	Other***	42 (16.6)
Net observed	Yes	2,057 (92.6)
Net hung up	Yes	1,816 (88.3)
Type of sleeping unit observed	Mat/carpet	996 (44.8)
	Mattress on the floor	880 (39.6)
	Mattress in Bed (wood or iron)	185 (8.3)
	Traditional mattress on the floor	26 (1.2)
	Traditional mattress in bed (wood or iron)	11 (0.5)
	Floor	124 (5.6)

/*Most common other net types were SafeNet, Panda Net 2.0.

**Most other origin of nets weas from other family members and friends.

***Most common other reason why nets were not in current use was refusal to use nets and reservation for visitors.

**Fig 2:**
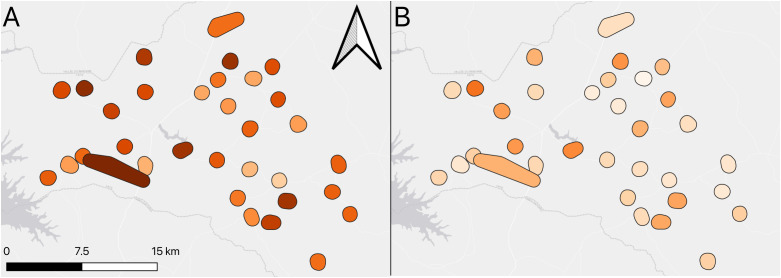
Map of LLIN coverage showing A) Household net ownership B) Household net access.

The proportion of households with at least one LLIN was 66.3% (95% CI: 59.4-73.2), varying from 28.0% to 100.0%, depending on the cluster. Less than a third of all households had sufficient nets for every 2 persons (27.8%; 95%CI: 22.6-33.1). This ranged by cluster from 2.0% to 62.0% (Fig 2).

### Long-lasting insecticidal net usage and associated factors in study participants

**Fig 3:**
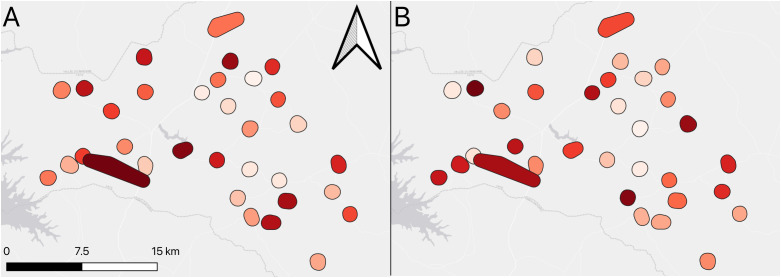
Map showing A) Bed net usage the previous night B) Malaria infection prevalence.

Slightly over half of study participants reported using LLINs the night before the survey (55.6%; 95%CI: 48.0-63.1), with a range from 20.0% to 94.0% by cluster (Fig 3). Amongst participants who lived in a household with at least one LLIN, LLIN usage the previous night was 83.9% (95%CI: 79.0-88.9%), whereas amongst those who lived in households with sufficient LLINs (based on 1 LLIN per 2 persons), LLIN usage the previous night was 91.4% (95%CI: 87.2-95.6).

**Fig 4:**
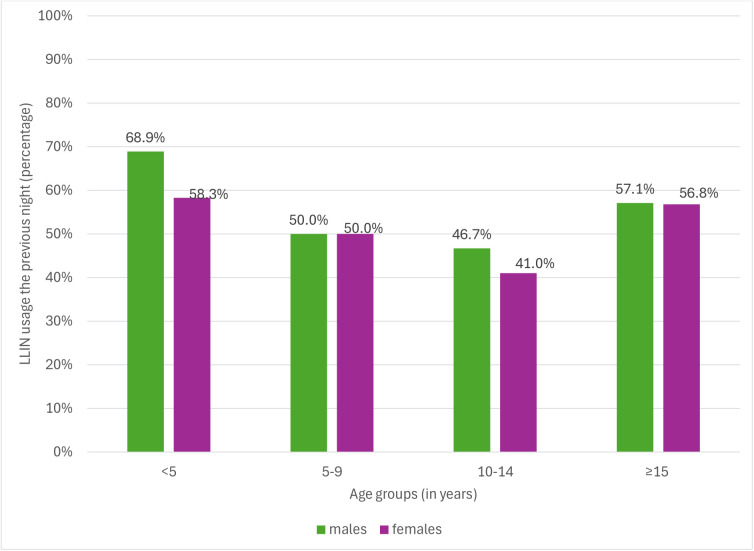
LLIN usage the previous night displayed by sex within each age group.

Bed net usage was lowest in children aged 10–14 years (Table 4). Age group and SES were associated with reported LLIN usage the previous night, even after adjustment for a priori confounders. Long-lasting insecticidal bed net usage was 50.0% (aOR: 0.50; 95% CI: 0.34-0.75) in 5–9 years, 44.3% (aOR: 0.41; 95%CI: 0.26-0.62) in 10–14 and 56.9% (aOR: 0.72; 95%CI: 0.53-0.98) in ≥15 years, compared to 63.2% in the under-fives. The odds of LLIN usage were lowest in the second (aOR: 0.63; 95%CI: 0.44-0.90) and middle wealth quintile (aOR: 0.69; 95%CI: 0.47-1.00) compared to the poorest quintile. There was no difference in LLIN usage between the poorest and richest quintiles (58.2% versus 58.5% respectively). Although there was no strong evidence for an association between sex and LLIN usage the previous night, Fig 4 shows that LLIN usage the previous night was lowest in females aged 10–14 years.

**Table 4:** Factors associated with LLIN usage the previous night.

				Univariate analyses		Multivariate analyses	
Factors	Categories	LLIN usage previous night, n(%) N = 929	No LLIN usage the previous night N = 743	Crude odds ratios (95% CI)^#^	p-value	Adjusted odds ratios (95% CI)*	p-value
Sex	Male	458 (56.5)	353 (43.5)	Ref.	0.58	Ref.	0.31
	Female	471 (54.7)	390 (45.3)	0.94 (0.76-1.17)		0.89 (0.72-1.11)	
Age group (in years)	<5	165 (63.2)	96 (36.8)	Ref.	0.0001	Ref.	0.0001
	5-9	116 (50.0)	116 (50.0)	0.50 (0.34-0.75)		0.52 (0.34-0.78)	
	10-14	82 (44.3)	103 (55.7)	0.41 (0.26-0.62)		0.40 (0.26-0.61)	
	≥15	566 (56.9)	428 (43.1)	0.72 (0.53-0.98)		0.73 (0.53-1.01)	
Wealth quintile^$^	Lowest	192 (58.2)	138 (41.8)	Ref.	0.005	Ref.	0.006
	Second	157 (47.1)	176 (52.9)	0.61 (0.43-0.87)		0.63 (0.44-0.90)	
	Middle	160 (49.4)	164 (50.6)	0.69 (0.48-0.99)		0.69 (0.47-1.00)	
	Fourth	213 (64.7)	116 (35.3)	1.08 (0.73-1.59)		1.10 (0.75-1.62)	
	Highest	192 (58.5)	136 (41.5)	0.91 (0.62-1.34)		0.94 (0.63-1.39)	
Religion	Christian	532 (52.9)	474 (47.1)	Ref.	0.40		
	Traditional	162 (58.7)	114 (41.3)	1.14 (0.81-1.60)			
	None	181 (58.0)	131 (42.0)	0.99 (0.72-1.39)			
	Other	54 (69.2)	24 (30.8)	1.56 (0.87-2.83)			
Closest distance between household and health facility (in km)^	<0.7	257 (46.6)	294 (53.4)	Ref.	0.33		
	0.7-5.1	337 (61.0)	215 (39.0)	1.55 (0.87-2.75)			
	>5.1	325 (58.9)	227 (41.1)	1.37 (0.64-2.93)			
Other insecticidal product use	No	720 (54.8)	593 (45.2)	Ref.	0.06		
	Yes	209 (58.2)	150 (41.8)	1.30 (0.99-1.71)			

^#^: univariate logistic regression models with random intercepts for clustering at cluster-level; *: logistic regression models with random intercepts for clustering at cluster-level adjusted for a priori confounders (sex, age group and wealth quintile); ^$^: 28 missing values for socio-economic status; ^: 17 missing values for closest distance from household to health facility; CI: confidence interval; p-values from Likelihood ratio tests.

### Malaria prevalence and associated risk factors

At the time of the survey, 199 study participants (11.9%) had a temperature ≥ 37.5^o^C, with a median age of 10 (IQR: 4.6-22.7) years. Amongst those with fever, malaria infection prevalence was 91.0% (n = 181), while the prevalence of malaria was 34.4% 506/1473) amongst those without a fever. A total of 603 participants (36.1%) had a history of fever in past 48h and/or current fever and/or headache. Amongst participants reporting symptoms suggestive of malaria, the prevalence of malaria infection was 74.0% (n = 446).

Overall, the prevalence of malaria infection in the study population was 41.1% (95%CI: 37.2-45.0), with variability by cluster from 21.6% to 76.0% (see Fig 3).

**Fig 5:**
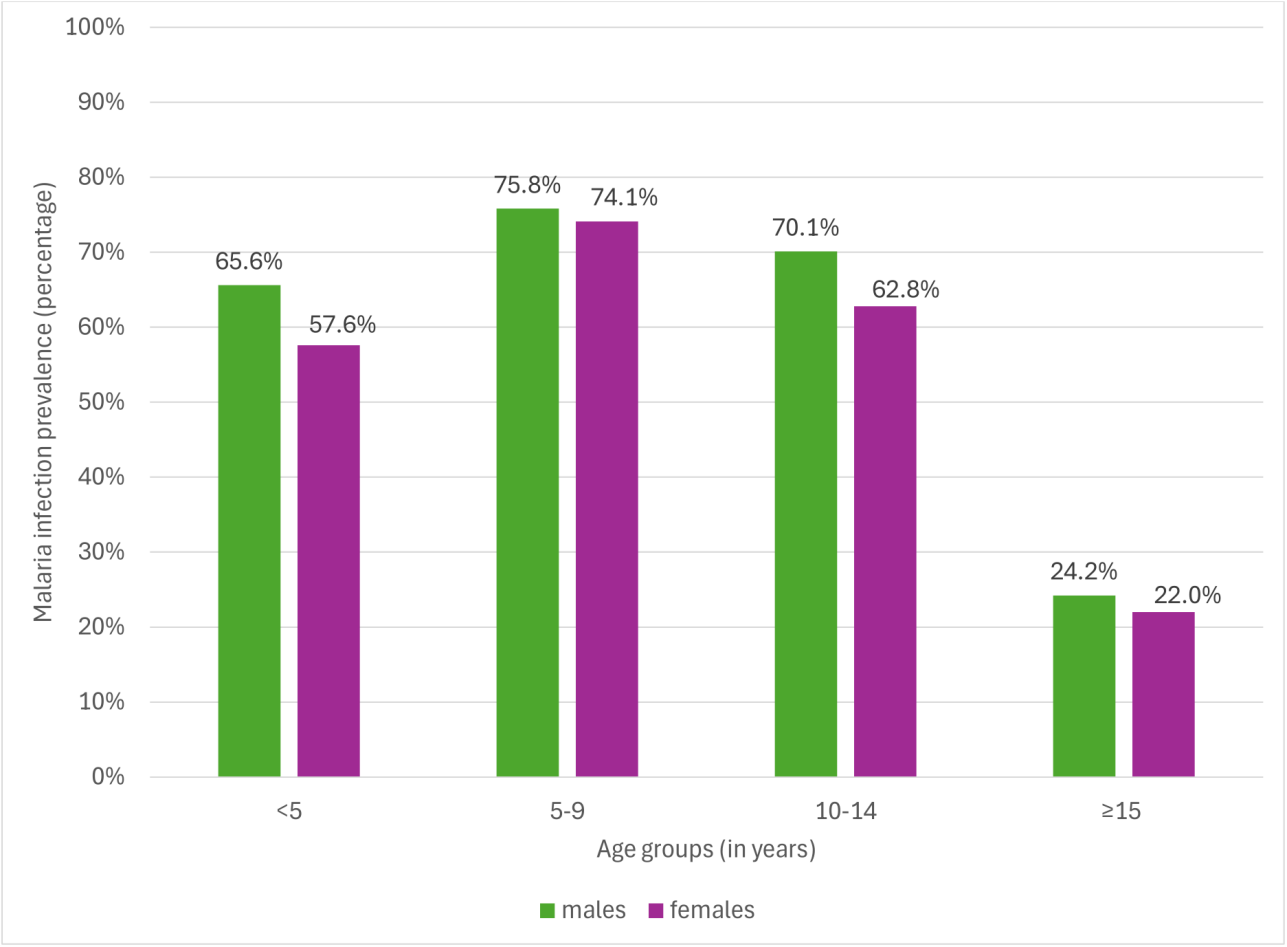
Malaria infection prevalence by sex within each age group.

Age group and LLIN usage the previous night were significant predictors of malaria infection, even after adjustment (Table 5). Malaria infection prevalence in 5–9, 10–14 and ≥15years was 75.0% (aOR: 1.86; 95%CI: 1.24-2.78), 67.0% (aOR:1.18; 95%CI: 0.78-1.80) and 23.0% (aOR: 0.17; 95%CI: 0.12-0.23), respectively, compared to 61.3% in under-fives. Fig 5 shows that males (75.8%) and females (74.1%) aged 5–9 years had the highest malaria infection prevalence. Those who reported using an LLIN the previous night had malaria prevalence of 38.5% (aOR: 0.74; 95%CI: 0.58-0.94), compared to 44.3% in those who did not use LLINs the previous night.

**Table 5:** Potential risk factors of malaria infection.

				Univariate analyses		Multivariate analyses	
Factors	Categories	Malaria positive, n(%) N = 687	Malaria negative, n(%) N = 985	Crude odds ratios (95% CI)^#^	p-value	Adjusted odds ratios (95% CI)^*^	p-value
Sex	Male	358 (44.1)	453 (55.9)	Ref.	0.02	Ref.	0.08
	Female	329 (38.2)	532 (61.8)	0.78 (0.64-0.95)		0.81 (0.65-1.02)	
Age group (in years)	<5	160 (61.3)	101 (38.7)	Ref.	<0.0001	Ref.	<0.0001
	5-9	174 (75.0)	58 (25.0)	1.98 (1.33-2.95)		1.86 (1.24-2.78)	
	10-14	124 (67.0)	61 (33.0)	1.27 (0.84-1.91)		1.18 (0.78-1.80)	
	≥15	229 (23.0)	765 (77.0)	0.17 (0.13-0.24)		0.17 (0.12-0.23)	
Wealth quintile^$^	Lowest	135 (40.9)	195 (59.1)	Ref.	0.35	Ref.	0.23
	Second	153 (46.0)	180 (54.0)	1.16 (0.84-1.62)		1.17 (0.80-1.70)	
	Middle	128 (39.5)	196 (60.5)	0.91 (0.65-1.27)		0.82 (0.56-1.21)	
	Fourth	127 (38.6)	202 (61.4)	0.85 (0.60-1.19)		0.63 (0.57-1.37)	
	Highest	132 (40.2)	196 (59.8)	0.89 (0.63-1.26)		0.79 (0.53-1.17)	
Religion	Christian	424 (42.2)	582 (57.8)	Ref.	0.15		
	Traditional	126 (45.6)	150 (54.4)	1.07 (0.79-1.43)			
	None	108 (34.6)	204 (65.4)	0.74 (0.55-0.99)			
	Other	29 (37.2)	49 (62.8)	0.75 (0.45-1.26)			
LLIN usage the previous night	No	329 (44.3)	414 (55.7)	Ref.	0.004	Ref.	0.015
	Yes	358 (38.5)	571 (61.5)	0.73 (0.59-0.90)		0.74 (0.58-0.94)	
Type of net used by participant	PermaNet2.0	247 (40.0)	370 (60.0)	Ref.	0.21		
	Other	111 (35.6)	201 (64.4)	0.82 (0.60-1.12)			
Duration of use of LLIN	<2 years	140 (41.2)	200 (58.8)	Ref.	0.61		
	2-3 years	150 (36.5)	261 (63.5)	0.84 (0.60-1.18)			
	>3 years	68 (38.2)	110 (61.8)	0.93 (0.61-1.42)			
Potential breeding sites around house	No	598 (41.3)	851 (58.7)	Ref.	0.89		
	Yes	89 (39.9)	134 (60.1)	0.98 (0.70-1.37)			
Other insecticidal product use	No	553 (42.1)	760 (57.9)	Ref.	0.18		
	Yes	134 (37.3)	225 (62.7)	0.84 (0.65-1.08)			
Closest distance between household and health facility (in km) ^	<0.7	242 (43.9)	309 (56.1)	Ref.	0.43		
	0.7-5.1	224 (40.6)	328 (59.4)	0.89 (0.64-1.26)			
	>5.1	211 (38.2)	341 (61.8)	0.78 (0.54-1.13)			
Altitude (in metres)	Very low	334 (41.2)	477 (58.8)	Ref.	0.87		
	Low	343 (40.6)	501 (59.4)	0.97 (0.72-1.31)			
Population density (in persons/km^2^)	Very low	364 (44.1)	462 (55.9)	Ref.	0.07		
	Low	313 (37.8)	516 (62.2)	0.77 (0.58-1.02)			
Nearest distance to a lake (in km)	<5.1	245 (44.5)	306 (55.5)	Ref.	0.57		
	5.1-8.6	221 (40.0)	331 (60.0)	0.87 (0.61-1.23)			
	>8.6	211 (38.2)	341 (61.8)	0.83 (0.58-1.18)			
NDVI	Low	217 (40.3)	322 (59.7)	Ref.	0.59		
	Moderate	228 (43.1)	301 (56.9)	1.19 (0.85-1.66)			
	High	232 (39.5)	355 (60.5)	1.10 (0.77-1.56)			

^$^: 28 missing values for socio-economic status; ^: 17 missing values for closest distance from household to health facility; ^#^: random effects univariate logistic regression models accounting for cluster-level clustering; ^*^: logistic regression models with random intercepts with clustering at cluster-level, adjusted for sex, age group, wealth quintile and LLIN usage the previous night; CI: confidence interval; NDVI: Normalized Difference Vegetation Index; p-values from Likelihood ratio tests.

### Long-lasting insecticidal net usage and malaria infection prevalence in school-aged children (5–14 years)

**Fig 6:**
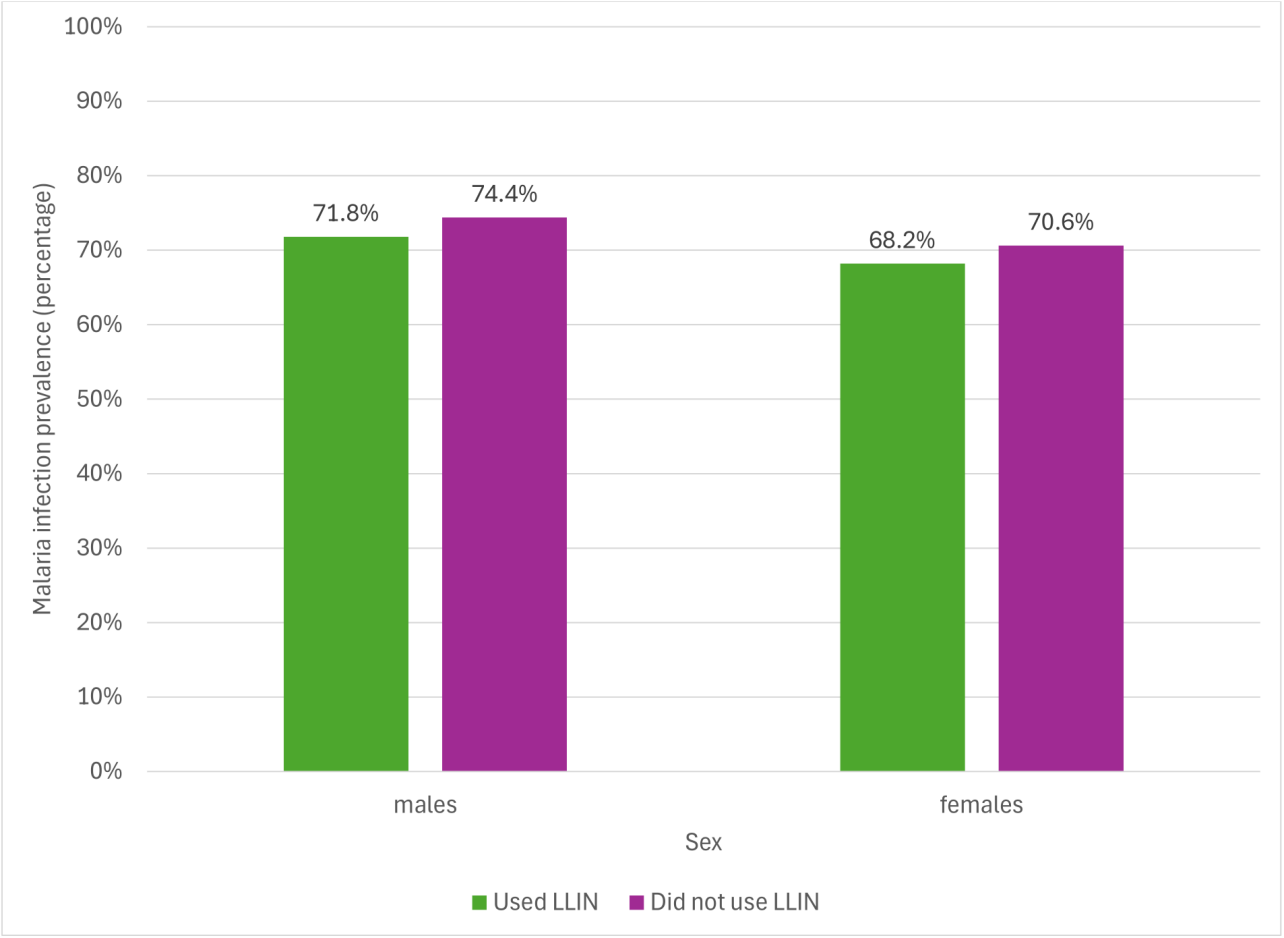
Malaria infection and LLIN usage by sex in school-aged children (5-14 years).

As shown in Table 6, reported LLIN usage the previous night was not associated with malaria infection prevalence (aOR: 0.80; 95%CI: 0.50-1.28) amongst school-aged children. Fig 6 shows that malaria infection prevalence amongst school-aged children who reported using LLINs the previous night was similar across both sexes.

**Table 6:** Association between bed net usage and malaria infection prevalence in school-age children (5-14 years).

				Univariate analyses		Multivariate analyses	
Factors	Categories	Malaria positive, n(%) N = 298	Malaria negative, n(%) N = 119	Crude odds ratios (95% CI)^#^	p-value	Adjusted odds ratios (95% CI)^$^	p-value
LLIN usage the previous night	No	159 (72.6)	60 (27.4)	Ref.	0.37	Ref.	0.34
	Yes	139 (70.2)	59 (29.8)	0.81 (0.51-1.29)		0.80 (0.50-1.28)	
Sex	Male	166 (73.1)	61 (26.9)	Ref.	0.35	Ref.	0.30
	Female	132 (69.5)	58 (30.5)	0.81 (0.52-1.26)		0.79 (0.50-1.24)	
Wealth quintile	Lowest	55 (72.4)	21 (27.6)	Ref.	0.62	Ref.	0.60
	Second	69 (75.8)	22 (24.2)	1.29 (0.61-2.71)		1.24 (0.58-2.62)	
	Middle	58 (70.7)	24 (29.3)	0.93 (0.44-1.96)		0.87 (0.40-1.86)	
	Fourth	49 (66.2)	25 (33.8)	0.71 (0.33-1.53)		0.68 (0.31-1.48)	
	Highest	63 (70.0)	27 (30.0)	0.86 (0.41-1.82)		0.83 (0.39-1.78)	
Religion	Christian	184 (71.0)	75 (29.0)	Ref.	0.19		
	Traditional	57 (81.4)	13 (18.6)	1.80 (0.89-3.64)			
	None	46 (66.7)	23 (33.3)	0.85 (0.45-1.59)			
	Other	11 (57.9)	8 (42.1)	0.56 (0.20-1.60)			
Potential breeding sites around house	No	263 (72.8)	98 (27.2)	Ref.	0.27		
	Yes	35 (62.5)	21 (37.5)	0.68 (0.34-1.35)			
Other insecticidal product use	No	242 (73.1)	89 (26.9)	Ref.	0.22		
	Yes	56 (65.1)	30 (34.9)	0.71 (0.41-1.23)			
Closest distance between household and health facility (in km)	<0.7	114 (74.0)	40 (26.0)	Ref.	0.79		
	0.7-5.1	90 (70.3)	38 (29.7)	0.87 (0.45-1.69)			
	>5.1	92 (70.2)	39 (29.8)	0.79 (0.40-1.57)			

^#^: random effects univariate logistic regression models accounting for cluster-level clustering; ^$^: logistic regression models with random intercepts with clustering at cluster-level, adjusted for sex and wealth quintile; CI: confidence interval; NDVI: Normalized Difference Vegetation Index; p-values from Likelihood ratio tests.

### Cluster-level correlation between LLIN access/usage and malaria infection prevalence

**Fig 7:**
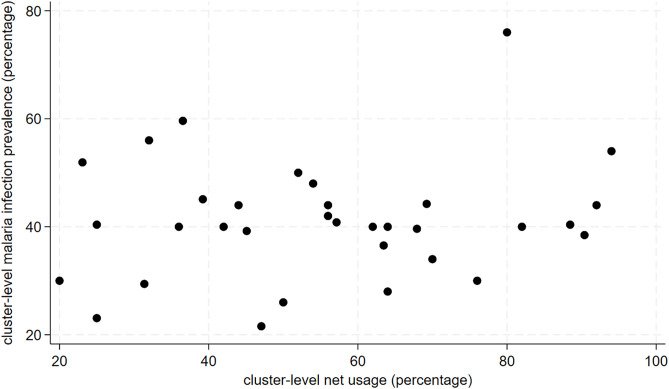
Scatterplot of cluster-level malaria infection prevalence and population bed net usage.

**Fig 8:**
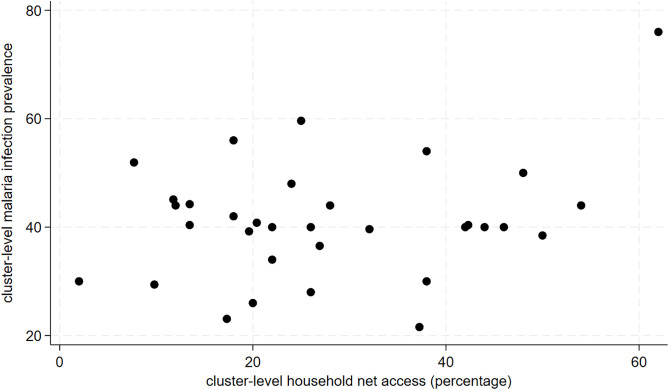
Scatterplot of cluster-level malaria infection prevalence and household LLIN access.

As shown in Figs 7 and 8, there was no correlation between cluster-level LLIN access or usage and malaria infection prevalence. An increase of 10% in cluster-level LLIN access and LLIN usage was associated with a 3.8% (95%CI: -0.9% to 8.6%; p-value: 0.11) and 3.1% (95%CI: -3.9% to 10.2%; p-value:0.37) increase in malaria infection prevalence, respectively albeit not statistically significant.

## Discussion

This baseline survey, conducted 2 years after the last mass distribution campaign and prior to a an LLIN trial, highlights persistent gaps in LLIN coverage and usage in central Côte d’Ivoire. Long-lasting insecticidal net usage was lowest amongst school-aged (5–14 years) children and amongst middle-income households. Malaria infection prevalence remained high at 41%, with younger school-aged children (5–9 years) exhibiting the greatest infection risk. Participants who reported sleeping under an LLIN the previous night had a modestly lower malaria prevalence than non-users.

Two years after a mass distribution, LLIN ownership (66%) and access (28%) fall well below the NMCP’s target of 100% ownership and 90% access by 2025 [3,4]. These values are similar to estimates in household LLIN ownership and access in Côte d’Ivoire in 2012 (67% and 32%, respectively) and 2018 (67.4% and 28.6%, respectively) and lower than in 2021 (72% and 51% respectively), showing a stagnation in progress made towards achieving universal LLIN coverage [2,3,25].This stagnation mirrors trends in other West African countries, where ownership has plateaued around 60% despite successive mass net distribution campaigns [26–28]. Similar declines in LLIN coverage two years post-campaign were also reported in Tanzania and Uganda, reflecting high net attrition and poor replacement rates [29,30]. The limited access observed here, despite reasonable ownership, underlines the persistent within-household gap—too few nets for household members—which remains one of the strongest predictors of non-use across endemic regions [30,31].

Self-reported LLIN usage in this study (56%) was moderate but comparable to past estimates from Côte d’Ivoire [3] as well as recent estimates from West Africa, such as Ghana, Nigeria and Benin [26–28]. This is below the NMCP’s ambition of 80% usage by 2025 [3].Encouragingly, amongst participants who lived in a household with sufficient LLINs, over 90% reported using them, indicating that access-rather than willingness-is the main barrier. This is consistent with multi-country analyses showing that when nets are available, most people tend to use them [32–34]. The large difference between ownership and access, and between access and use, points to the need for improved quantification of household needs during campaign planning and for behavioural “top-up” strategies in the years between campaigns.

Disparities in LLIN usage by age and SES were notable. LLIN use was lowest among school-aged children, particularly girls aged 10–14 years (41%)—a group also showing high malaria infection prevalence. Similar patterns have been observed across sub-Saharan Africa, where school-aged children consistently report lower LLIN use than younger children or adults, attributed it to lower caregiver supervision and changing sleep arrangements as children grow older [35–37]. As this age group forms a key reservoir for malaria transmission [38], interventions targeting school-aged children—such as school-based net distribution, community education, or expansion of seasonal malaria chemoprevention, could have high public health impact. The finding that middle-income households were less likely to use LLINs than poorer or wealthier ones, while unusual, may be due to perceived lower malaria risk or may be because these householders have alternative prevention methods. This finding suggests that communication strategies should not exclusively target the poorest but also address complacency among households transitioning out of poverty.

The prevalence of malaria infection among febrile individuals was 91%, compared to 34% among afebrile participants, supporting fever as a useful but imperfect proxy for malaria infection in high-transmission settings. Malaria infection prevalence was high (41%). This is similar to estimates from south-central Côte d’Ivoire at 46.0% and 56.6% during the transmission season of 2010 and 2011 respectively [39], suggesting no progress towards a reduction in malaria infection prevalence. Malaria infection prevalence in children under the age of 5 years (61%) was the highest recorded across 19 sub-Saharan Africa countries, including Côte d’Ivoire in 2021 [40]. The persistence of high prevalence in Côte d’Ivoire likely reflects both intense transmission and widespread pyrethroid resistance in local vector populations [3]. Considerable variability in malaria infection prevalence was observed across clusters (22–76%) which could reflect local differences in transmission intensity influenced by ecological, genetic and behavioural factors [41,42]. Similar spatial heterogeneity has been documented in Benin, Ghana, Togo and Burkina Faso, suggesting that surveillance and control interventions could be geographically adaptive, rather than uniformly applied [40] Although sleeping under an LLIN reduced the odds of malaria infection by about one-quarter, infection prevalence among users remained substantial (38.5%). This residual transmission underscores the limits of pyrethroid-only LLINs in high-resistance settings. Trials in Benin, Tanzania and Uganda have demonstrated higher protection with next-generation LLINs incorporating piperonylbutoxide or chlorfenapyr [7–9] Given that Côte d’Ivoire’s 2021 campaign distributed standard deltamethrin nets, these findings support the NMCP’s recent shift toward deploying dual-active ingredient nets nationwide.

From a public health perspective, the high infection rates in school-aged children and persistent inequities in LLIN access call for renewed targeting of this age group. Integrating school-based vector control, scaling up community-led surveillance, and prioritising equitable LLIN replacement could substantially reduce the malaria burden. Strengthening behavioural change communication and gender-sensitive strategies is also critical to sustain LLIN use across all household members.

### Strengths and limitations

This study had several strengths, including a very high response rate and minimal missing data, which reduces the risk of bias arising from incomplete observations. Nonetheless, as with any observational cross-sectional study, residual confounding from unmeasured or imperfectly measured factors cannot be fully excluded. In addition, although our sample size was sufficient to estimate main associations with reasonable precision, it provided limited statistical power to detect interaction effects (e.g., between sex and socio-economic status or between age and sex), meaning that results should be interpreted with caution.

## Conclusion

Two years after the last mass distribution, LLIN ownership, access, and use in central Côte d’Ivoire remain below national and global targets, with school-aged children and middle-income households disproportionately underserved. The high malaria prevalence despite moderate LLIN use underscores the need for next-generation LLINs, tailored behavioural strategies, and integrated approaches that combine vector control with preventive treatment for high-risk groups. Strengthening these efforts will be essential for Côte d’Ivoire to move closer to malaria elimination goals.

## Supporting information

S1 Checklist Inclusivity in global research questionnaire. (DOCX)

## References

[1] World malaria report 2023. Geneva: World Health Organization. 2023.

[2] Institut National de la Statistique-INS, I C F. Enquête démographique et de santé de Côte d’Ivoire, 2021. Rockville, Maryland, USA: INS/Côte d’Ivoire et ICF. 2022.

[3] U.S. President’s Malaria Initiative Cote d’Ivoire Malaria Profile. U.S. President’s Malaria Initiative. 2023.

[4] U.S. President’s Malaria Initiative Côte d’Ivoire Malaria Operational Plan FY 2022. U.S. President’s Malaria Initiative. 2022.

[5] The Global Fund, Roll Back Malaria. Malaria Matchbox Tool: An Equity Assessment Tool to Improve the Effectiveness of Malaria Programs. Geneva, Switzerland: The Global Fund. 2020.

[6] State of inequality: HIV, tuberculosis and malaria. Geneva: World Health Organization. 2021.

[7] Mosha JF, Kulkarni MA, Lukole E, Matowo NS, Pitt C, Messenger LA, et al. Effectiveness and cost-effectiveness against malaria of three types of dual-active-ingredient long-lasting insecticidal nets (LLINs) compared with pyrethroid-only LLINs in Tanzania: a four-arm, cluster-randomised trial. Lancet. 2022;399(10331):1227–41. doi: 10.1016/S0140-6736(21)02499-5 35339225 PMC8971961

[8] Accrombessi M, Cook J, Dangbenon E, Sovi A, Yovogan B, Assongba L, et al. Effectiveness of pyriproxyfen-pyrethroid and chlorfenapyr-pyrethroid long-lasting insecticidal nets (LLINs) compared with pyrethroid-only LLINs for malaria control in the third year post-distribution: a secondary analysis of a cluster-randomised controlled trial in Benin. Lancet Infect Dis. 2024;24(6):619–28. doi: 10.1016/S1473-3099(24)00002-1 38401551

[9] Staedke SG, Gonahasa S, Dorsey G, Kamya MR, Maiteki-Sebuguzi C, Lynd A, et al. Effect of long-lasting insecticidal nets with and without piperonyl butoxide on malaria indicators in Uganda (LLINEUP): a pragmatic, cluster-randomised trial embedded in a national LLIN distribution campaign. Lancet. 2020;395(10232):1292–303. doi: 10.1016/S0140-6736(20)30214-2 32305094 PMC7181182

[10] Sarfo JO, Amoadu M, Kordorwu PY, Adams AK, Gyan TB, Osman AG. Malaria amongst children under five in sub-Saharan Africa: a scoping review of prevalence, risk factors and preventive interventions. Eur J Med Res. 2023;28:80. 36800986 10.1186/s40001-023-01046-1 PMC9936673

[11] Chilot D, Mondelaers A, Alem AZ, Asres MS, Yimer MA, Toni AT, et al. Pooled prevalence and risk factors of malaria among children aged 6-59 months in 13 sub-Saharan African countries: A multilevel analysis using recent malaria indicator surveys. PLoS One. 2023;18(5):e0285265. doi: 10.1371/journal.pone.0285265 37256889 PMC10231787

[12] Anjorin S, Okolie E, Yaya S. Malaria profile and socioeconomic predictors among under-five children: an analysis of 11 sub-Saharan African countries. Malar J. 2023;22(1):55. doi: 10.1186/s12936-023-04484-8 36788541 PMC9927033

[13] Garley AE, Ivanovich E, Eckert E, Negroustoueva S, Ye Y. Gender differences in the use of insecticide-treated nets after a universal free distribution campaign in Kano State, Nigeria: post-campaign survey results. Malar J. 2013;12:119. doi: 10.1186/1475-2875-12-119 23574987 PMC3635971

[14] Olapeju B, Choiriyyah I, Lynch M, Acosta A, Blaufuss S, Filemyr E, et al. Age and gender trends in insecticide-treated net use in sub-Saharan Africa: a multi-country analysis. Malar J. 2018;17(1):423. doi: 10.1186/s12936-018-2575-z 30428916 PMC6234545

[15] Babalola S, Adedokun ST, McCartney-Melstad A, Okoh M, Asa S, Tweedie I, et al. Factors associated with caregivers’ consistency of use of bed nets in Nigeria: a multilevel multinomial analysis of survey data. Malar J. 2018;17(1):280. doi: 10.1186/s12936-018-2427-x 30071875 PMC6071383

[16] Quaresima V, Agbenyega T, Oppong B, Awunyo JADA, Adu Adomah P, Enty E, et al. Are malaria risk factors based on gender? A mixed-methods survey in an urban setting in Ghana. Trop Med Infect Dis. 2021;6:161. 34564545 10.3390/tropicalmed6030161 PMC8482108

[17] UNDP. Discussion paper: Gender and malaria, making the investment case for programming that addresses the specific vulnerabilities and needs of both males and females who are affected by or at risk of malaria. 2015.

[18] Sih C, Protopopoff N, Koffi AA, Ahoua Alou LP, Dangbenon E, Messenger LA, et al. Efficacy of chlorfenapyr-pyrethroid and piperonyl butoxide-pyrethroid long-lasting insecticidal nets (LLINs) compared to pyrethroid-only LLINs for malaria control in Côte d’Ivoire: a three group, cluster randomised trial. Trials. 2024;25(1):151. doi: 10.1186/s13063-024-07969-2 38419075 PMC10900640

[19] Le departement de tiebissou. https://rezoivoire.net/ivoire/villes-villages/426/le-departement-de-tiebissou.html

[20] Sih C, Assi SB, Talbot B, Dangbenon E, Kulkarni MA, Koffi AA, et al. Evaluation of household coverage with long-lasting insecticidal nets in central Côte d’Ivoire. Malar J. 2025;24(1):104. doi: 10.1186/s12936-025-05335-4 40158121 PMC11955107

[21] Danielson JJ, Gesch DB. Global multi-resolution terrain elevation data 2010 (GMTED2010): U.S. Geological Survey Open-File Report 2011–1073, 26. 2011. 2011.

[22] EROS Visible Infrared Imaging Radiometer Suite (eVIIRS) Global NDVI. https://www.usgs.gov/centers/eros/science/usgs-eros-archive-vegetation-monitoring-eviirs-global-ndvi

[23] WorldPop. https://hub.worldpop.org/doi/10.5258/SOTON/WP00674

[24] Messager ML, Lehner B, Grill G, Nedeva I, Schmitt O. Estimating the volume and age of water stored in global lakes using a geo-statistical approach. Nat Commun. 2016;7:13603. doi: 10.1038/ncomms13603 27976671 PMC5171767

[25] Johns Hopkins Center for Communication Programs. Survey of Determinants of Malaria-Related Behavior in Côte d’Ivoire. 2019. https://breakthroughactionandresearch.org/wp-content/uploads/2020/02/Cote-dIvoire-MBS-Report-EN-2019SEP17.pdf

[26] U.S. President’s Malaria Initiative. U.S. President’s Malaria Initiative Nigeria Malaria Profile PMI (FY-2024). 2023. https://mesamalaria.org/wp-content/uploads/2025/04/NIGERIA-Malaria-Profile-PMI-FY-2024.pdf

[27] U.S. President’s Malaria Initiative. Ghana malaria profile PMI (FY-2024). 2023. https://mesamalaria.org/wp-content/uploads/2025/04/GHANA_Malaria_Profile_PMI_FY_2024.pdf

[28] U.S. President’s Malaria Initiative. Benin malaria profile PMI (FY-2024). 2023. https://mesamalaria.org/wp-content/uploads/2025/04/BENIN-Malaria-Profile-PMI-FY-2024.pdf

[29] Massue DJ, Moore SJ, Mageni ZD, Moore JD, Bradley J, Pigeon O, et al. Durability of Olyset campaign nets distributed between 2009 and 2011 in eight districts of Tanzania. Malar J. 2016;15:176. doi: 10.1186/s12936-016-1225-6 26993981 PMC4797150

[30] Gonahasa S, Maiteki-Sebuguzi C, Rugnao S, Dorsey G, Opigo J, Yeka A, et al. LLIN Evaluation in Uganda Project (LLINEUP): factors associated with ownership and use of long-lasting insecticidal nets in Uganda: a cross-sectional survey of 48 districts. Malar J. 2018;17(1):421. doi: 10.1186/s12936-018-2571-3 30424775 PMC6234693

[31] Kuetche MTC, Tabue RN, Fokoua-Maxime CD, Evouna AM, Billong S, Kakesa O. Prevalence and risk factors determinants of the non-use of insecticide-treated nets in an endemic area for malaria: analysis of data from Cameroon. Malar J. 2023;22(1):205. doi: 10.1186/s12936-023-04510-9 37407962 PMC10320958

[32] Ekusai-Sebatta D, Arinaitwe E, Mpimbaza A, Nankabirwa JI, Drakeley C, Rosenthal PJ, et al. Challenges and opportunities for use of long-lasting insecticidal nets to prevent malaria during overnight travel in Uganda: a qualitative study. Malar J. 2021;20(1):283. doi: 10.1186/s12936-021-03811-1 34174892 PMC8235645

[33] Fernández Montoya L, Alafo C, Martí-Soler H, Máquina M, Malheia A, Sacoor C, et al. An evaluation of LLIN ownership, access, and use during the Magude project in southern Mozambique. PLoS One. 2023;18(3):e0282209. doi: 10.1371/journal.pone.0282209 36972236 PMC10042371

[34] Bertozzi-Villa A, Bever CA, Koenker H, Weiss DJ, Vargas-Ruiz C, Nandi AK, et al. Maps and metrics of insecticide-treated net access, use, and nets-per-capita in Africa from 2000-2020. Nat Commun. 2021;12(1):3589. doi: 10.1038/s41467-021-23707-7 34117240 PMC8196080

[35] Basiru A, Alozieuwa UB, Aboh MI, Usman UY, Nwaefulu ON, Okeke OP, et al. Utilization of insecticide-treated nets for malaria prevention among children in Africa: a systematic review and meta-analysis. Malar J. 2025;24(1):358. doi: 10.1186/s12936-025-05599-w 41131520 PMC12548209

[36] Buchwald AG, Walldorf JA, Cohee LM, Coalson JE, Chimbiya N, Bauleni A, et al. Bed net use among school-aged children after a universal bed net campaign in Malawi. Malar J. 2016;15:127. doi: 10.1186/s12936-016-1178-9 26928321 PMC4770676

[37] Storey JD, Babalola SO, Ricotta EE, Fox KA, Toso M, Lewicky N, et al. Associations between ideational variables and bed net use in Madagascar, Mali, and Nigeria. BMC Public Health. 2018;18(1):484. doi: 10.1186/s12889-018-5372-2 29642883 PMC5896159

[38] Cohee LM, Opondo C, Clarke SE, Halliday KE, Cano J, Shipper AG, et al. Preventive malaria treatment among school-aged children in sub-Saharan Africa: a systematic review and meta-analyses. Lancet Glob Health. 2020;8(12):e1499–511. doi: 10.1016/S2214-109X(20)30325-9 33222799 PMC7721819

[39] Epidemiology of malaria in the Taabo health and demographic surveillance system, south-central Côte d’Ivoire. https://pubmed.ncbi.nlm.nih.gov/26739224/10.1186/s12936-015-1076-6 PMC4704401 26739224

[40] Yitageasu G, Worede EA, Alemu EA, Tigabie M, Birhanu A, Angelo AA, et al. Malaria prevalence and its determinants across 19 sub-Saharan African countries: a spatial and geographically weighted regression analysis. Malar J. 2025;24(1):305. doi: 10.1186/s12936-025-05573-6 41029676 PMC12487587

[41] Oumbouke WA, Pignatelli P, Barreaux AMG, Tia IZ, Koffi AA, Ahoua Alou LP. Fine scale spatial investigation of multiple insecticide resistance and underlying target-site and metabolic mechanisms in Anopheles gambiae in central Côte d’Ivoire. Sci Rep. 2020;10:15066. 32934291 10.1038/s41598-020-71933-8 PMC7493912

[42] Oumbouke WA, Tia IZ, Barreaux AMG, Koffi AA, Sternberg ED, Thomas MB, et al. Screening and field performance of powder-formulated insecticides on eave tube inserts against pyrethroid resistant Anopheles gambiae s.l.: an investigation into “actives” prior to a randomized controlled trial in Côte d’Ivoire. Malar J. 2018;17(1):374. doi: 10.1186/s12936-018-2517-9 30348154 PMC6196564

